# An effective and safe innovation for the management of vault prolapse

**DOI:** 10.1186/1750-1164-4-6

**Published:** 2010-10-19

**Authors:** Rajiv Mahendru

**Affiliations:** 1 Department of Obs & Gyn, Institute M.M.I.M.S.R., Mullana, Ambala, Haryana, India

## Abstract

**Objective:**

Considering the great variety of techniques and disagreement about the ideal route, there is a need for a simple, safe and effective method for the management of vault prolapse.

**Study Design:**

51 cases of post- hysterectomy vault prolapse: 45 following vaginal and 6 after total abdominal hysterectomy were treated surgically by anterior abdominal wall colpopexy with autogenous rectus fascia strips.

**Results:**

Except for minor complaints like vomiting, fever and urinary retention in 3.92% cases each (n = 2 each), no major complications were encountered. Moreover, no recurrence, thus far, on follow-up.

**Conclusion:**

Using autogenous rectus fascia strips in anterior abdominal wall colpopexy is not only simple, cheap and effective method of treating apical prolapse but is also devoid of any serious complications as described with other techniques.

## Introduction

Post-hysterectomy apical (vault) prolapse is referred to as the descent of the vaginal vault/cuff scar below a point that is 2 cm less than the total vaginal length above the plane of the hymen [1]. This condition is shown to follow 11.6% of hysterectomies performed vaginally for prolapse and 1.8% of those performed abdominally for other indications[2]. Several surgical procedures have been described for the treatment of vault prolapse and given the available evidence of complications and limitations of each, it is difficult to recommend one technique over the other[3]. Regarding vault suspension to the anterior abdominal wall, enough studies are not available to assess its efficacy.

## Materials and methods

In the period between May 2002 and July 2008, 51 cases of vaginal vault prolapse were treated with the innovative technique described hereunder.88.23% (n = 45) had had undergone vaginal hysterectomy for genital prolapse and the remaining 11.77% (n = 6) had apical prolapse developing after abdominal hysterectomy for the indication of leiomyoma uteri in 4 cases and dysfunctional uterine bleeding in the other 2 cases. The mean time elapsed from the previous surgery was 29 months with the shortest being 13 months and the longest being 50 months. Two of the cases of vaginal hysterectomy group had undergone prior surgery for the vault prolapse- one had sacrocolpopexy 13 months back, and the other had some vaginal procedure 27 months back, record of which was not available but both these patients developed recurrence.

Mean age of our patients was 59.3 years with the youngest & oldest being 37.0 & 69.5 years respectively.

Written informed consent was taken and the Departmental Ethical Committee approved the study.

Based on the quality of life, complaints, history and clinical examination- a questionnaire proforma was filled for each patient. Nine of the patients had stress incontinence (all after vaginal hysterectomy).

Previous history of one patient was that of Pulmonary Tuberculosis for which she had had a full course of anti-tubercular treatment and at the time of admission was symptom free. Prior to taking up the patient for surgery, presence of chronic cough & chronic constipation complaints were clearly addressed to.

Clinical examination was re-conducted for the presence of enterocoele (n = 9), cystocoele (n = 17), rectocoele (n = 23), genuine stress incontinence (n = 9).

Initially, bladder cathetrisation with 16 French indwelling foley's and tight vaginal packing done pre-operatively.

Technique employed included: transverse skin incision (Pfannenstiel) about 8 cm long at the level of anterior superior iliac spine. Rectus sheath was exposed by separating the subcutaneous adipose tissue from its surface for the entire length of the incision and about 2 cm in breadth.

About 1 cm longitudinal incision was given on the rectus sheath in the midline and laterally this incision extended transversely about 3-4 cm on either side superiorly as well as inferiorly beyond the lateral margin of the rectus muscle. Now that two strips of the rectus fascia were devised (*figure- 1 and 2*) with free medial margins which were secured with silk no.1 with the thread kept long on both the sides.

**Figure 1:**
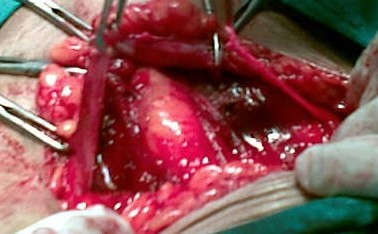
**RECTUS FASCIA STRIPS**.

**Figure 2:**
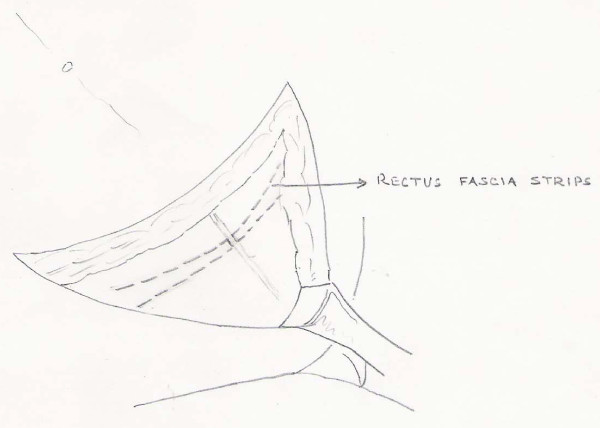
**Diagramatic representation showing Rectus fascia strips**.

Peritoneal cavity was approached in the usual way followed by abdominal packing. Vault (*figure- 3*) identified (easy because of initial vaginal packing) and held with Allis' forceps at both of its angles. Peritoneum between these Allis' forceps over the vault was incised.

**Figure 3:**
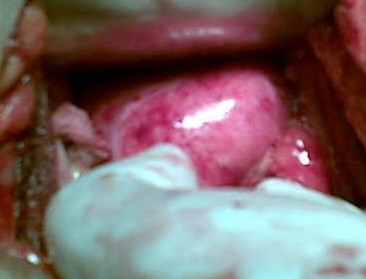
**vault (easily identified because of pre-op. vaginal packing)**.

**Figure 4:**
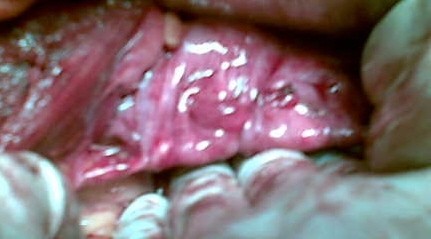
**pulled-up vault after fixation of the rectus fascia strips**.

**Figure 5:**
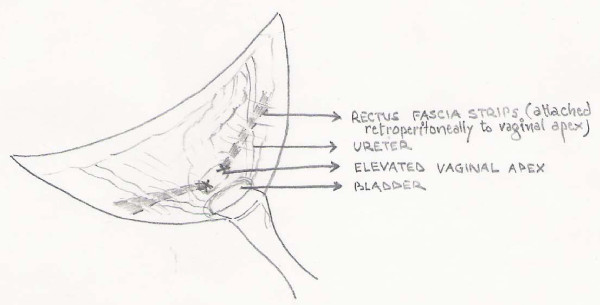
**Diagramatic representation showing fixation of the rectus fascia strips**.

A long curved artey forceps was used to make a track retroperitoneally, parallel to the erstwhile round ligament exercising utmost care so as to avoid ureter on the way. On reaching lateral to the rectus muscle the medial margin of the ipsilateral already carved rectus sheath strip was held and brought to the angle of the vault ensuring that no twisting occurred. Similar procedure was performed on the opposite side. Once done, the silk threads kept long earlier, were tied on to the vault with optimal tension (*figure-4 and 5*) so that rectus fascia strips after fixation were neither too taut nor so lax as to defeat the purpose of surgery.

Re-peritonisation accomplished by suturing the incised peritoneum on the vault with chromic catgut no. 1-0.

Patients having enterocoele were treated with Moschowitz operation. In those nine patients having stress incontinence, concomitant pubovaginal sling repair was carried out.

Abdomen was closed in layers after securing haemostasis and removing the sponge pack.

Sub-cuticular sutures were applied in all the cases.

Vaginal packing done prior to surgery, removed.

In cases with rectocele, perineorrhaphy with levator myorrhaphy was done followed by vaginal packing for 24 hrs in such cases.

Intra venous fluids continued for about 24 hrs and injectable antibiotics for 36 hours. Oral fluids were offered after the bowel sounds appeared (on an average after 24 hours). Patient was made ambulatory 24-36 hours following surgery.

Catheter was removed after 24 hours except in patients who underwent anti-incontinence repair alongwith, where it was taken out after 5 days post-operatively.

### Observations

Mean operating time was approximately 52 minutes: those cases in whom only anterior abdominal wall colpopexy was done the procedure was accomplished in 47 minutes but in those cases in whom additional surgical procedures were required like

Moschowitz/Burch/perineorrhaphy, extra 15 minutes were taken but operating duration never extended beyond 70 minutes.

Intraoperative difficulties encountered were not much annoying: 7 cases (13.73%) had omental and 3 (5.88%) had small gut adhesions on the vault.

Amount of intra operative blood loss was between 200 to 450 ml (more loss in cases where perineorrhaphy was done alongwith); nevertheless, no blood transfusion was required in any of the cases neither during surgery nor post-operatively.

In the post-operative period two patients (3.92%) had vomiting on the 2^nd ^day, another two (3.92%) had fever. 25.49% (n = 13) patients felt slight tightness in the abdomen but not amounting to discomfort of concern.

Two patients (3.92%) who had simultaneous urinary anti-incontinence operation had urinary retention following removal of the indwelling catheter on the 5^th ^post-operative day and had to be re-catheterised for another 48 hrs and toning-up of the bladder done.

One patient (1.96%) had bleeding from the perineorrhaphy wound on removal of the vaginal pack but not too alarming to be of concern. 42 (82.35%) patients were discharged 3 days after surgery, eight of those nine cases (15.68%) who had concomitant pubo-vaginal sling for stress incontinence were discharged 5 days after surgery; one of these patients had urinary retention following catheter removal and was discharged after 3 more days. Another patient had retention of urine after discharge from the hospital but was re-admitted for 72 hrs.

Sub-cuticular abdominal wound stitches were removed on the7th day of surgery in all the patients.

At discharge, per-speculum examination was conducted and the success criteria considered was that on straining the vault, which was prolapsing pre-operatively, was seen being pulled up/retracted.

39 patients (76.47%) are under regular follow-up, (longest follow-up-5 yrs 3 months and the patient with the shortest follow-up of 14 months), 12 patients (23.53%) were lost after initial one or two follow-ups. Another questionnaire proforma was filled after one year of regular postoperative visits of these 39 patients. All the patients on regular follow-up confirm the success of the procedure as none of these have reported recurrence of the symptoms.

## Discussion

The incidence of vault prolapse is uncertain but appears to be increased five fold after vaginal hysterectomy[4].

Conservative measures include pelvic floor exercises and different type of pessaries.There is no evidence to suggest whether pelvic floor exercises are helpful in vault prolapse[5]. Conservative measures may be used to treat this condition in women unfit for surgery or those who require symptomatic relief while awaiting surgery[4].

Correction of apical defect remains a surgical challenge: suspension of the vaginal apex is the keystone of surgical repair for pelvic organ prolapse.

There are many surgical procedures to treat post-hysterectomy vaginal vault prolapse and may be either through vaginal or abdominal approach[6].

Anterior and posterior vaginal wall repair along with obliteration of the enterocoele sac are inadequate for post-hysterctomy vaginal vault prolapse: this standard repair operation does not support the vaginal vault and risks vaginal narrowing and shortening and thus dyspareunia[7].

The established surgical options lie between a vaginal sacrospinous fixation or abdominal procedure such as sacrocolpopexy or vault suspension operation[4]. Vaginal repair often results in a narrowed & shortened vagina with diminished function[8]. Moreover, posterior vaginal sling experience in elderly patients yields poor results[9]. Complications of sacrospinous fixation included-blood loss, bladder injury, rectovaginal haematoma and vaginal pain and that of sacrocolpopexy- blood loss, bladder injury, incisional hernia, mesh rejection, wound infection[10]. In another study sacrospinous fixation was associated with significantly more intraoperative blood loss, longer cathetrisation and hospital stay with more sexual dysfunction[11]. Moreover, it has a high failure rate[10,12] with significantly higher incidence of recurrent vault prolapse and recurrent stress incontinence in the vaginal group[13].

Jenkins, Stuart and McCoubrie in 2008[14] had successful use of rectus sheath tendon flap in 20 patients of vaginal vault prolapse with minimal complications. Anterior abdominal wall colpopexy using autogenous strips of rectus fascia to repair post-hysterectomy prolapse of the vaginal vault has the advantage of preservation of a physiologically useful vagina, its caliber and depth with no reported recurrence[15].

A comparative study of abdominal colpopexy using rectus fascia and sacral fixation for the treatment of prolapsed vagina following hysterectomy concluded that the abdominal approach yields better results[16].

Although, augmentation by foreign material is not altogether a new concept, the introduction of commercial kits make mesh procedures more standardized but not necessarily technically more easy to perform[17]. Recently, a study involving hitching the vault with a synthetic tape inserted along the lines of the round ligaments & fastening it to the external oblique aponeurosis reported encouraging results[18] but use of this synthetic mesh was reported to be associated with infection[19].

Patients who are additionally incontinent, an anti-incontinence procedure such as Burch colposuspension or pubo-vaginal sling is usually performed at the same time[19]. Role of prophylactic surgery for occult stress incontinence is unclear[20].

It is imperative that all pelvic floor deficiencies should be identified to enable surgical repair of these significant and potential defects[21].

Of late, the transvaginal use of the uterosacral-cardinal ligament complex is gaining increasing popularity in the surgical treatment of posthysterectomy vault prolapse[22] but studies indicate apossibility of neural compromise after uterosacral ligament suspension[23]. According to Barber et al, [24] intraoperatively ureteral occlusion was noted in 11%, symptomatic prolapse developed in 10%and reoperation had to be performed.

The great variety of techniques described indicate that there is disagreement about the ideal route or procedure to be adopted[6].

## Conclusion

No doubt, greater awareness at the time of original hysterectomy may be the better solution in reducing the incidence of vault prolapse, nevertheless, anterior abdominal wall colpopexy using autogenous strips of rectus fascia is a simple operation,to repair post-hysterectomy prolapse of the vaginal vault providing the desirable result of preserving a physiologically useful vagina with near normal caliber and depth; moreover, no recurrence in follow-up.

From the above study conclusion drawn is that this technique of anterior abdominal wall colpopexy is a safe operation associated with low morbidity, long standing good results with patient satisfaction. and can be recommended for all women of apical prolapse including those who are sexually active but comparative studies may be conducted.

## Conflict of interest

the authors declare that they have no competing interests.
